# Newly Generated Cells in the Dentate Gyrus Differentially Respond to Brief Spatial Exploration and Forced Swim in Adult Female Balb/C Mice

**DOI:** 10.1155/2018/4960869

**Published:** 2018-05-22

**Authors:** Gerardo Bernabé Ramírez-Rodríguez, María del Ángel Ocaña-Fernández, Leonardo Ortiz-López

**Affiliations:** Laboratory of Neurogenesis, Division of Clinical Research, National Institute of Psychiatry “Ramón de la Fuente Muñiz”, Calzada México-Xochimilco 101, 14370 Mexico City, Mexico

## Abstract

Neurogenesis in the hippocampus is influenced by several factors including external stimuli. In addition to their involvement in learning and memory processes, newborn neurons of the dentate gyrus (DG) buffer against the effects of stress. Although the response of these cells to environmental stimuli has been shown, the age of the cells that respond to a brief spatial exploration or a stressful situation produced by forced-swim stress in adult female Balb/C mice is still unknown. Here, we investigated the activation of newborn neurons after three (IdU) or six weeks (CldU) postlabelling with the expression of Arc in the same mice but exposed to different environmental stimuli. Mice housed in standard conditions showed an increase in the activation of CldU-labelled cells after two exposures to a brief spatial exploration but no increase in the activation of IdU-labelled cells compared with the control group. Additionally, we analysed neuronal activation in the DG of mice housed in standard conditions and further exposed to forced-swim stress. We found a decreased activation of IdU-labelled cells in mice exposed to forced-swim stress with increase number of CldU-labelled cells. Our results suggest that based on their time postlabelling, newly generated hippocampal neurons show a different response to several environmental stimuli.

## 1. Introduction

The generation of newborn neurons in the DG of the hippocampus during adulthood is regulated by several external stimuli (i.e., [1–4]). Among them, voluntary physical activity and environmental enrichment have proven to have beneficial effects on neuroplasticity in, but not limited to, the hippocampus (i.e., [1–4]). Additionally, hippocampal neurogenesis is negatively regulated by stressful paradigms such as acute stress, psychosocial stress, or unpredictable chronic mild stress (i.e., [4, 5]).

Interestingly, newly generated hippocampal neurons play an important role in the processing of information related to spatial memory (i.e., [6–8]). Additionally, the presence of more neurons in the DG is indicative for their participation in the adaptation to complex environments, particularly because newborn neurons can help buffer the effects of stress (i.e., [9, 10]).

The enrolment of newly generated neurons in information processing has been studied by examining the expression of immediate early gene (IEG) products such as c-Fos, zif268, and activity-regulated cytoskeletal-associated protein (Arc) (i.e., [11–19]). The IEGs are transiently and rapidly induced in response to several stimuli. These genes and their products have relevant roles in several processes including brain development, learning, and long-term synaptic plasticity (for review, see [20]).

Additionally, depending on the age of the newborn neurons, they have been suggested to be able to be involved not only in processing specific information such as spatial learning, spatial memory retrieval, contextual memory, and spatial exploration but also in processing a stressful experience in rodents (i.e., [11–21]).

In this sense, hippocampal newly generated cells of ages ranging between 28 and 42 days old in C57Bl6 mice previously exposed to voluntary physical activity show a decrease in activation after exposure to a cold forced-swim stress [17]. Moreover, no difference was found in cells 7 and 84 days old after forced-swim exposure in mice preexposed to ENR conditions [22].

Despite the information related to the activation of newborn cells in the DG, there is still a discrepancy, from our point of view, in the age of newborn hippocampal cells that are activated in response to different environmental stimuli. Thus, we hypothesized that depending on their age, newborn neurons will differentially respond to environmental stimuli. Then, we determined the age and the environmental stimuli to which newly generated cells in the DG respond in adult female Balb/C mice, a strain of mice with low baseline adult neurogenesis [23] but with the capability to reflect the regulation of adult hippocampal neurogenesis (i.e., [24–26]). We exposed standard housed mice to different configurations of spatial exploration or to forced-swim stress as was previously reported for rats and mice [11] to explore the response of newly generated cells, at 3 or 6 weeks postlabelling, to the environmental stimuli.

## 2. Materials and Methods

### 2.1. Animals

Thirty Balb/C female mice were used in this study. They were held in standard laboratory cages with 12 h light/12 h dark cycles at a temperature of 23 ± 1°C in the animal facilities of the National Institute of Psychiatry Ramón de la Fuente Muñiz. Mice had access to food and water ad libitum and were left to acclimate to their environment until animals reached ten weeks of age. All institutional and legal regulations regarding animal ethics and handling were followed for in vivo experiments (IACUC: CEI/C/009/2013).

### 2.2. CldU and IdU Labelling

All animals were injected intraperitoneally (ip) with two analogues of thymidine: 5-chloro-2′-deoxyuridine (CldU) and 5-iodo-2′-deoxyuridine (IdU) (MP Biomedicals, Santa Ana, CA, USA). On the first day of housing, all mice received a single injection of CldU (42.5 mg/kg), and on the twenty-first day of housing, all mice received one single IdU injection (57.5 mg/kg) [27]. Six weeks later, after the beginning of the housing protocol, mice experienced a behavioural task. With this procedure, we identified newborn cells at 21 days postlabelling (IdU) or 42 days postlabelling (CldU).

### 2.3. Experiments

#### 2.3.1. Experiment 1

Mice were maintained in standard housing conditions, and on day 42, animals experienced a behavioural task. Animals were distributed into four groups: (1) mice decapitated directly from their cages (control, CTL); (2) mice that explored environment A once (A); (3) mice that explored environment A twice, each separated by 20 min (A + A); or (4) mice that explored two different environments, A and B, each separated by 20 min (A + B). The exploration time in each environment was 5 min. Mice were adapted to handling and transportation procedures for 3 minutes each day for seven consecutive days before the behavioural task. The day before environmental explorations, animals were left undisturbed.

Environment A was a square open box (30 × 30 cm) with 8.5 cm high walls, two of which were covered with black foamy paper. The box was divided into nine squares; two of them were covered by brown corrugated paper. Environment A was placed in a rectangular room (2 × 1.5 m) with spatial stimuli on the walls.

Environment B was a rectangular open box (25 × 35 cm) with 8.5 cm high walls; two of which were covered with blue foamy paper. The box was divided in 8 rectangular sections; three of which were covered with green corrugated paper. Exposure to environment B was done in another room of similar size but with different spatial stimuli on the walls. Both environments, A and B, were different; however, they had a similar level of complexity [11]. In each exploration session, mice were lifted and randomly placed in the centre of the grids of the corresponding explored environment for 15 seconds for a total period of five minutes [11, 21]. Fifty-five minutes after the first environmental exposure, mice were sacrificed.

#### 2.3.2. Experiment 2

To test the effect of stress on Arc protein expression in newly generated cells, mice were exposed or not exposed to forced-swim stress on day 42. Mice were maintained in standard housing conditions for six weeks. Mice were divided into two groups: (1) mice housed in standard conditions and sacrificed directly from their cages (No-FS) or (2) mice housed in standard conditions followed by exposure to forced-swim stress (FS).

The forced-swim stress paradigm, also known as Porsolt's test, is a test that measures an animal's response to a stressful situation in which they are forced to swim in a cylinder from which there is no opportunity for escape. Mice were gently placed in a cylinder (18 cm in diameter) filled with water maintained at room temperature to a depth of approximately 15 cm. Each mouse was tested for 6 min; the first 2 min served as a habituation period. The time spent immobile during the last 4 min was recorded. Immobility was assessed when a mouse was floating in the water without struggling and only making movements necessary to keep its head above water. After testing, each mouse was gently dried and returned to their cage covered by an absorbent paper towel [28, 29]. Fifty-four minutes later, mice were sacrificed by transcardial perfusion [30].

### 2.4. Tissue Preparation

Brains were immersed in 4% *p*-formaldehyde and kept for 7 days. Then, brains were cryoprotected before they were cut into 40 *μ*m coronal sections on a sliding microtome (Leica, Buffalo Grove, IL, USA). The sections were stored at 4°C in cryoprotective solution containing 25% ethylene glycol and 25% glycerol in 0.05 M phosphate buffer [30].

### 2.5. Immunohistochemistry and Immunofluorescence

Sections were stained following the free-floating immunohistochemistry method [30]. To visualize the expression of Arc protein, IdU, and CldU, we used the peroxidase method, as previously described [30]. For IdU and CldU immunodetection, brain slices were pretreated with 2 N HCl for 30 min at 37°C followed by 2 washes in 0.1 M borate buffer (pH 8.5) for 10 min each. The antibodies used were the rat anti-BrdU (CldU; 1 : 500; Accurate), mouse anti-BrdU (IdU; 1 : 500; BD Pharmingen), and rabbit anti-Arc antibody (1 : 5000; Synaptic Systems) to measure neuronal activation in the DG. Secondary antibodies were biotinylated and added for 2 additional hours of incubation at room temperature. All secondary antibodies were from Jackson ImmunoResearch Laboratories (West Grove, PA, USA). Finally, the cells that were positive for the different markers were visualized using the ABC-DAB system (Vector Labs; Burlingame, CA, USA). Sections were clarified and mounted with Neo-Mount medium (Merck Millipore; Naucalpan, Estado de México, México).

Identification of newly formed cells in the DG of adult mice that expressed the Arc protein was performed by double immunofluorescence (CldU/Arc or IdU/Arc). The primary antibodies were mouse anti-IdU, rat anti-CldU, and rabbit anti-Arc. The fluorophore-coupled secondary antibodies were anti-rat FITC, anti-mouse FITC, and anti-rabbit TRITC. All secondary antibodies were raised in donkey and diluted 1 : 200 [31]. Sections were mounted in polyvinyl alcohol with diazabicyclo-octane as an antifading agent (PVA-DABCO).

### 2.6. Cellular Quantification and Neuronal Activation Colabelling

The number of IdU-, CldU-, and Arc-labelled cells was determined in series of every 6th section from all animals. Positive cells were counted using a 40x objective throughout the rostrocaudal extent of the granular cell layer on a light microscope (Leica) (Bregma −1.34 to −3.20). Counting was done as previously described with a modified optical fractionator method. The cells appearing in the uppermost focal plane were not counted [30]. The estimate of the total number of cells was calculated from the resulting numbers through multiplication by 6.

Analysis of newly formed cells activated in the DG was performed in a one-in-twelve series of sections from all animals. The number of IdU- and CldU-labelled cells coexpressing the Arc protein was quantified by confocal microscopy (TCS SP2, Leica, and on a Zeiss LSM 510 Meta, Zeiss, Ciudad de México, México) in a sequential scanning mode to avoid cross-bleeding between channels. Double labelling was confirmed by three-dimensional reconstructions of z-series covering the entire nucleus or cell in question. IdU-Arc or CldU-Arc cells were calculated from the percentages of the respective labelled cells and the total number of IdU- or CldU-labelled cells [25, 29, 30].

### 2.7. Statistical Analysis

Analysis was performed using SigmaStat 3.1 software (Systat Software Inc., Chicago, IL, USA). The results are presented as the mean ± standard error of the mean; SEM. Mean differences between two groups were analysed using unpaired Student's *t*-test. Mean differences among groups were analysed using a one-way ANOVA followed by Student-Newman-Keuls post hoc test. Differences were considered statistically significant at *p* ≤ 0.05.

## 3. Results

### 3.1. Newborn Cells Generated in the Dentate Gyrus during Housing Conditions

**Figure 1:**
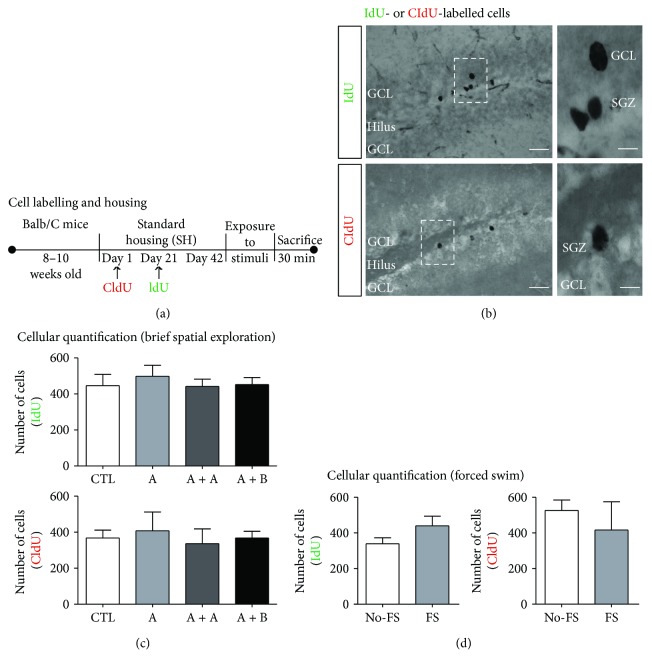
The experimental design to visualize newborn cells in the dentate gyrus of adult mice. (a) Female Balb/C mice were kept under standard housing (SH) conditions. Mice received one single injection of CldU (day 1) and another injection of IdU at day 21. Then, mice were housed for an additional 22 days in SH conditions. (b) Representative micrographs of IdU- or CldU-labelled cells in the SH group. High-power images exhibit the presence of IdU- or CldU-labelled cells. Labels identify granule cell layer (GCL), subgranular zone (SGZ), and the hilus. Scale bars are equal to 40 and 10 *μ*m. (c) Quantification of IdU- and CldU-labelled cells was done in mice decapitated directly from their cages (control, CTL), mice that explored environment A once (a), mice that explored environment A twice, each separated by 20 min (A-A), or 4) mice that explored two different environments, (a) and (b), each separated by 20 min (A–B). *n* = 5 per group. Data are expressed as the mean ± SEM of the absolute number of Arc-cells, and comparisons in (c) were performed with a one-way ANOVA followed by Student-Newman-Keuls post hoc test. Also in the second experiment, cellular quantification was done in mice housed in standard conditions and sacrificed directly from their cages and not exposed to forced swim (No-FS) or mice housed in standard conditions followed by exposure to forced-swim stress (FS). *n* = 5 per group. Data are expressed as the mean ± SEM of the absolute number of Arc cells, and comparisons in (d) were performed with an unpaired Student *t*-test.

To visualize IdU or CldU newborn cells in the same animals housed in standard conditions, we injected two analogues of thymidine (Figure 1(a)); we quantified IdU- and CldU-labelled cells in the DG (Figure 1(b)) of mice exposed to brief spatial exploration (CTL, A, A + A, or A + B) or in mice of the second experiment exposed to forced-swim stress. Mice of the first experiment did not show significant differences in the number of IdU- (CTL = 445 ± 63, A = 498 ± 60, A + A = 441 ± 41, and A + B = 451 ± 39; F3, 19 = 0.25; *p* = 0.85) or CldU-labelled cells (CTL = 367 ± 43, A = 408 ± 104, A + A = 336 ± 82, and A + B = 367 ± 38; F3, 19 = 0.16; *p* = 0.91) (Figure 1(c)). Moreover, mice of the second experiment exposed to forced-swim stress (FS) did not show significant differences in the number of IdU- or CldU-labelled cells compared with the not-exposed-to-FS group (No-FS) (IdU: No − FS = 339 ± 33, FS = 439 ± 54, *p* = 0.16; CldU: No − FS = 523 ± 59, FS = 414 ± 158, *p* = 0.54). Thus, these results showed newborn cells at 3 or 6 weeks after administration of different analogues of thymidine in the same mice.

### 3.2. Distribution of Arc in Cells of the Granular Cell Layer of the Dentate Gyrus

**Figure 2:**
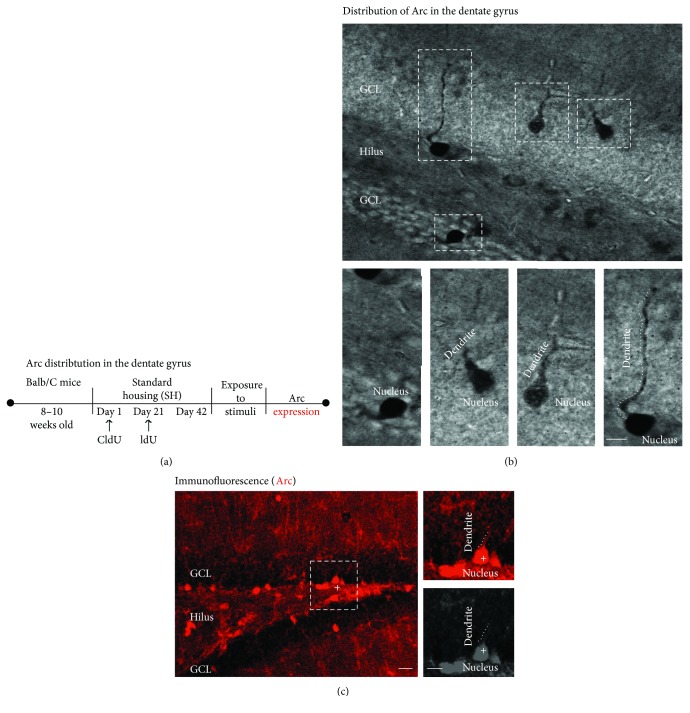
Arc expression in the dentate gyrus of adult mice. (a) Experimental design. (b) Arc-positive cells are present in the dentate gyrus. Bright field also shows the granule cell layer (GCL), hilus (H), and molecular layer (ML). High-power images exhibit the presence of Arc in the nucleus, and Arc labelling is present in the dendrites projecting to the ML. The scale bar in (b) is equal to 10 *μ*m (c) Also, the distribution of Arc is shown by immunofluorescence. Scale bars in (c) are equal to 20 and 10 *μ*m.

First, we studied Arc distribution in cells of the granular cell layer of the DG (Figures 2(a) and 2(b)). Arc cells showed a well-stained nucleus within oval or round somas. Also, some Arc-positive cells exhibited a short dendrite. Moreover, some Arc-positive cells showed one primary dendrite with one branching point or a well-defined dendrite projecting to the direction of and reaching the molecular layer. Similar distribution of Arc was observed by immunofluorescence (Figure 2(c)). In accordance with the images, we confirmed that Arc distribution was not limited to the nucleus of immunoreactive cells of the GCL of the DG.

### 3.3. Arc Expression Increased in the Dentate Gyrus after Spatial Exploration or Forced-Swim Stress

**Figure 3:**
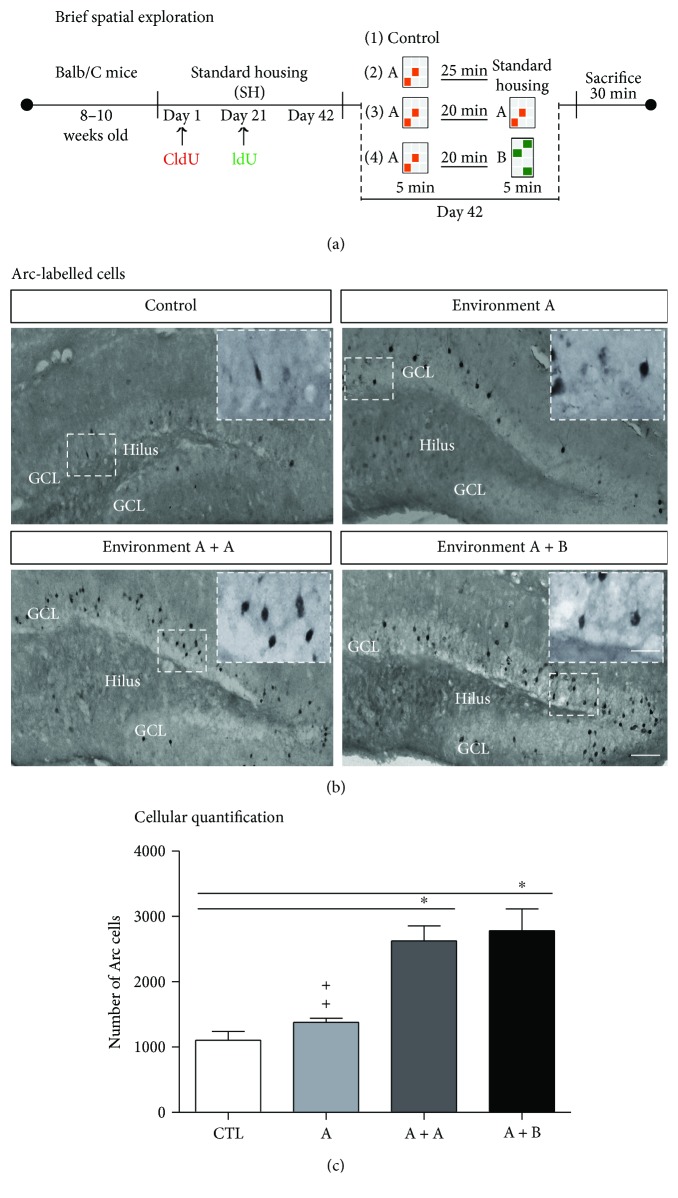
Neuronal activation in the dentate gyrus of adult mice after brief spatial exploration. (a) Experimental design. Female Balb/C mice kept under standard housing conditions were exposed to environment A, A + A, or A + B for 5 minutes as is described in Materials and Methods. (b) Representative micrographs of Arc-labelled cells in mice exposed to the different environments are shown. Scale bar in B is equal to 100 *μ*m. Insets correspond to high-power images of Arc-positive cells. The scale bar in the inserts is equal to 40 *μ*m. (c) Quantification of Arc-labelled cells showed more in the A + A and A + B groups than in the control (CTL) group. Asterisks indicate *p* = 0.001. Crosses indicate a significant difference between A and A + A (*p* = 0.006) or A and A + B (*p* = 0.002). Data are expressed as the mean ± SEM of the absolute number of Arc-cells, and comparisons in (c) were performed with a one-way ANOVA followed by Student-Newman-Keuls post hoc test. *n* = 4 − 5 per group.

**Figure 4:**
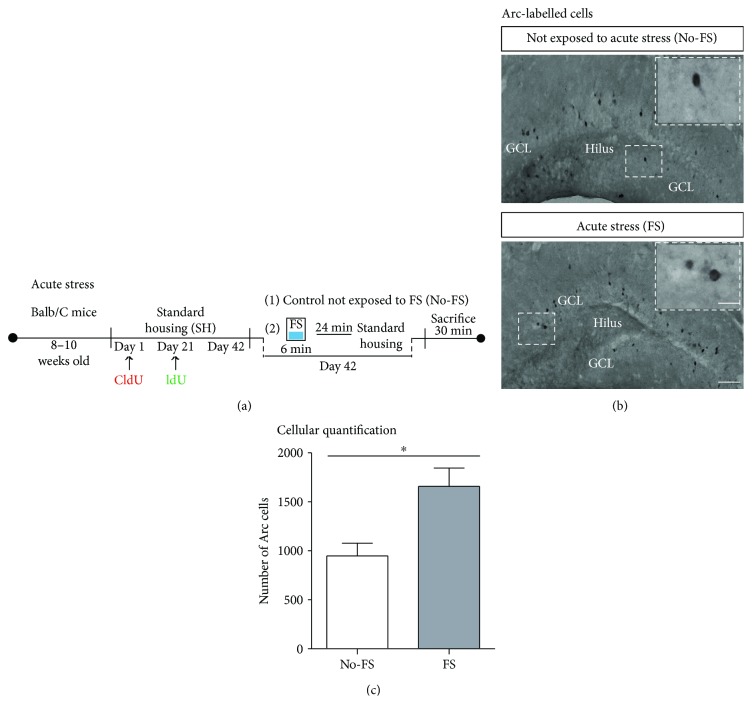
Neuronal activation in the dentate gyrus of adult mice after forced-swim exposure. (a) Experimental design. Female Balb/C mice kept under standard housing conditions were exposed to forced-swim stress (FS) for 5 minutes as described in Materials and Methods. (b) Representative micrographs of Arc-labelled cells in mice exposed to forced-swim are shown. The scale bar in B is equal to 100 *μ*m. Insets correspond to high-power images of Arc-positive cells. The scale bar in the insets is equal to 20 *μ*m. (c) Quantification of Arc-labelled cells showed more in the FS group than in the No-FS group. Asterisk indicate *p* = 0.035. Data are expressed as the mean ± SEM of the absolute number of Arc-cells, and comparisons in (c) were performed with unpaired Student's *t*-test. *n* = 4 − 5 per group.

On day 42, mice experienced a brief spatial exploration task or were exposed to the forced-swim stress (Figures 3(a) and 4(a)). To assess the activation of granule cells in the DG in response to each task, we identified the cells labelled with Arc.

Analysis of experiment 1 showed significant differences among the groups (F3, 19 = 15.53, *p* < 0.001; Figures 3(b) and 3(c)). The post hoc test did not show a difference between the *CTL* group (1106 ± 130 cells) and mice exposed to environment A a single time (1379 ± 63 cells; *p* = 0.709). However, there was a significant increase in the number of Arc-labelled cells in mice exposed to environment A twice *(2624 ± 231 cells)* compared with that in the control group (*p* = 0.001) or with that in mice exposed to environment A once (*p* = 0.006). Similarly, we found significant differences between group A + B (2780 ± 335 cells) and the control group (*p* = 0.001) and group A (*p* = 0.002). However, there was no significant difference between the mice in the A + A and A + B groups (*p* = 0.961).

Moreover, analysis of Arc-labelled cells in experiment 2 (Figure 4(a)), in which mice were kept in standard conditions for 42 days followed by forced-swim exposure, revealed that the forced-swim condition showed a statistically significant effect on the expression of the Arc protein in the granule cells of the DG (Figures 4(b) and 4(c); No − FS = 948 ± 129 cells, FS = 1658 ± 186 cells; *p* = 0.035).

### 3.4. IdU-Labelled Cells Were Not Affected but CldU-Labelled Cells Increased Their Coexpressing Arc after Spatial Exploration

**Figure 5:**
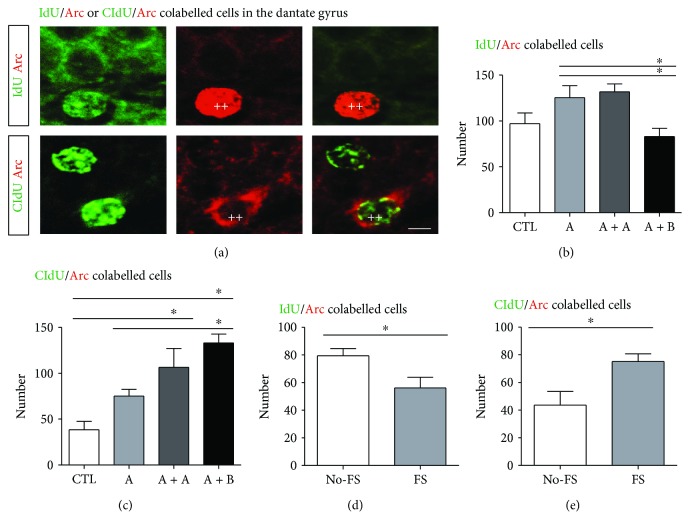
Activation of newborn cells in the dentate gyrus of adult mice. (a) Representative micrographs of IdU/Arc- and CldU/Arc-labelled cells are shown. IdU or CldU (green), Arc (red), and the merged micrographs are shown. The scale bar is equal to 10 *μ*m. (b–c) Quantification of IdU- and CldU-activated (Arc) cells in mice exposed to brief spatial exploration is shown. The number of newborn activated (Arc) cells was calculated by multiplying the ratio of newborn activated IdU or CldU cells with the absolute number of IdU- or CldU-labelled cells, respectively. (b) The number of IdU/Arc-labelled cells increased in mice exposed to environment A once or twice in comparison to that in mice subsequently exposed to environments A and B. Asterisks indicate *p* < 0.05. However, an increase in the number of CldU/Arc-labelled cells was found in mice exposed to environment A twice or in mice subsequently exposed to environment (a and b) in comparison to that in mice exposed to environment A or the control group (c). Asterisks indicate *p* = 0.03 and *p* = 0.001, respectively. Data are expressed as the mean ± SEM, and comparisons were performed with a one-way ANOVA followed by Student-Newman-Keuls post hoc test. *n* = 4 − 5 per group. (d, e) The number of IdU/Arc-labelled cells was significantly lower compared to mice exposed to forced swim (FS) than that found in mice not exposed to forced swim (No-FS). However, CldU/Arc-labelled cells were significantly higher in mice exposed to forced swim (FS). The asterisk in (d) indicates *p* = 0.047. The asterisk in (e) indicates *p* = 0.031. *n* = 4 − 5 per group. Data are expressed as the mean ± SEM, and comparisons were performed with unpaired Student's *t*-test.

To obtain information about the activation of IdU- and CldU-labelled cells after behaviour, we analysed cells coexpressing the Arc protein as indicative of neuronal activation in the DG (Figure 5(a)). Analysis of IdU/Arc-labelled cells in the mice from experiment 1 reflected that the number of newborn cells at three weeks post-IdU labelling was not significantly increased in the DG of mice exposed once (125 ± 13 cells) or twice to environment A (131 ± 8 cells) compared to the cells found in mice of the control group (97 ± 11 cells). Moreover, no differences were found in mice exposed to environments A and B (83 ± 8 cells) when compared with the control mice. The one-way ANOVA yielded the following values: F3, 19 = 4.59; *p* = 0.016; Figure 5(b)).

In addition, the analysis of CldU/Arc protein-labelled cells revealed that mice exposed once to environment A (75 ± 7 cells) showed a trend toward an increase in the number of CldU/Arc-labelled cells compared to control mice (38 ± 9 cells). However, mice exposed to environment A twice (106 ± 20 cells) or mice exposed to environment A followed by environment B (133 ± 9 cells) showed activation at six weeks post-CldU labelling of cells in the DG compared with the control group (*p* = 0.03 and *p* = 0.001, respectively; Figure 5(c)). Additionally, we found more CldU/Arc cells in mice exposed to environment A followed by environment B than in mice exposed once to environment A (*p* = 0.03), but no differences were found between groups A + A and A + B. The one-way ANOVA yielded the following values: F3, 19 = 7.082; *p* = 0.0046.

### 3.5. The Coexpressing Arc of IdU-Labelled Cells Decreased but That of CldU-Labelled Cells Increased after Forced-Swim Stress

The analysis of experiment 2 showed differences in the number of IdU/Arc-labelled cells among the different groups (No − FS = 79 ± 5, FS = 56 ± 7; *p* = 0.047Figure 5(d)). However, the analysis of CldU/Arc-labelled cells revealed that mice exposed to forced-swim stress had more activated cells at six weeks post-CldU labelling in the DG (No − FS = 43 ± 9, FS = 75 ± 5; *p* = 0.031; Figure 5(e)).

## 4. Discussion

**Figure 6:**
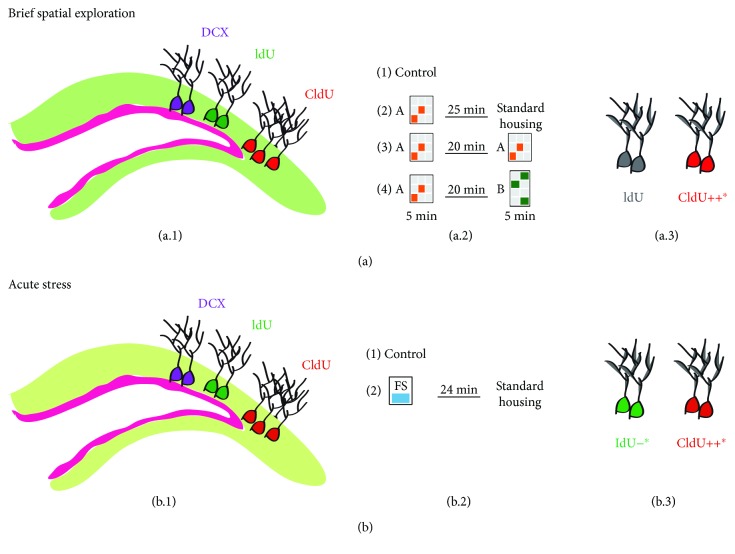
Representation of the results derived from the present study. (a) Brief spatial exploration. (a.1) Illustration representing the newly generated cells analysed in our study that corresponds to IdU (green)- and CldU (red)- labelled cells. (a.2) Illustration representing the experimental design indicating the three groups tested with a brief spatial exploration and the control group. The illustration also indicates the duration of the explorations and the amount of time between both environment configurations. (a.3) The representative drawings indicate that newborn cells (CldU, 42 days postlabelling) mainly respond to brief spatial exploration. (b) Forced-swim stress (acute stress). (b.1) Illustration representing the newly generated cells analysed in our study that corresponds to IdU (green)- and CldU (red)- labelled cells. (b.2) The illustration represents the experimental design showing the two groups tested in experiment 2. The illustration also indicates the duration of the test and the time until the mice were sacrificed. (b.3) The representative drawings indicate that newborn cells (CldU, 42 days postlabelling) mainly respond to forced-swim exposure whereas IdU-labelled cells (21 days postlabelling) decrease activation in mice housed in standard conditions but exposed to forced swim.

This study showed that newborn neurons of the DG exhibit differential activation after three or six weeks postlabelling in adult female Balb/C mice housed in standard conditions further exposed to a spatial exploration paradigm or to forced-swim stress (Figure 6).

### 4.1. Expression of Arc in the Dentate Gyrus of Adult Mice

Newborn hippocampal neurons are key elements that process information related to spatial exploration (i.e., [8]). In this regard, several studies have explored activation of newborn hippocampal neurons and have shown evidence of the enrolment of these cells in the processing of several types of information (i.e., [11–19]). Here, we exposed mice housed in standard conditions to a paradigm of spatial exploration that has been used mainly in rats and that assures standardization of the amount of space explored by each rodent (i.e., [11, 21]). In this sense, during spatial exploration, rodents are known to create a representation of the environment by activating neuronal circuits in the hippocampus (i.e., [11]). Thus, neuronal activation in the granular cell layer of the dentate gyrus has been studied by the expression of IEGs including Arc, c-Fos, or zif268 (i.e., [11–19]).

In this sense, Arc is distributed in the nucleus, dendrites, and synapses of activated neurons [31, 32]. Interestingly, neuronal activation is observed in granular cells distributed in the inner, middle, or outer layers of the granular cell layer of the dentate gyrus (i.e., [13]). Previous studies have reported that a “small population of granular neurons in the dentate gyrus expressed Arc after exploration suggesting a sparse dentate gyrus code for spatial information” (i.e., [11, 13]).

In this sense, our mice exposed to the spatial exploration paradigm showed different levels of neuronal activation measured with the expression of Arc.

Contrary to what was previously reported in rats (i.e., [11, 13]), we did not find an increase in activation in mice exposed to a new environment once. This result is similar to previous data showing neuronal activation evaluated with zif268 expression in male mice exposed to the Morris water maze after 42 days of standard housing conditions [12]. Moreover, the exposure of adult female Balb/C mice to two identical or different environments similarly increased neuronal activation, which is different than what has been shown in rats (i.e., [11, 33]). Those reports have indicated different levels of neuronal activation determined with the expression of the messengers coding for Arc and zif268. Additionally, reexposure of rats to the same environment resulted in activation of the same neural ensemble for each exposure, but the exposure of rats to two different environments resulted in activation of a newborn population of neurons in response to the exposure to the second environment [11, 33], an effect that we did not find in our study. Our data indicate that mice exposed to the same environment twice or to two different environments showed an increase in neuronal activation compared to mice with or without a single exposure. These data suggest that, independently of the configuration of the second environment, there is a neuronal population with the capability to respond to the stimuli provided by the second environment. Similar to the neuronal activation presented in spatial exploration paradigms [11, 33], exposure to a stressful situation has also been described to involve the codification of information allowing the animal to exhibit a better coping strategy in the second exposure [34]. Here, mice exposed to the forced-swim stress, which is a stressful situation for rodents [28], after normal housing showed increased levels of neuronal activation. Our results are in line to a previous study in which male C57Bl6 mice exposed to forced swim show increase neuronal activation in the dentate gyrus [17].

### 4.2. Expression of Arc in Newborn Cells in the Dentate Gyrus of Adult Mice after Spatial Exploration

Newly generated hippocampal neurons are able to functionally integrate into neuronal circuits, which can be analysed by the expression of IEGs, which are related to plasticity and memory. Thus, several studies have reported expression of c-Fos, zif268, or Arc in response to behavioural stimuli in newborn hippocampal neurons (i.e., [14, 21, 35, 36]). Here, we found a trend towards a decrease in activation of granule cells after 3 weeks post-IdU labelling in mice exposed to two different environments; however, an increase in activation was evident in newborn cells analysed after 6 weeks post-CldU labelling in mice exposed to the environments once or twice, with a higher increase in mice exposed to different environments. The analysis of CldU/Arc-colabelled cells reflected similar results to those found in rats (i.e., [11]), suggesting that, in the mouse hippocampus, there are different ensembles of newborn cells with the capability of being activated in response to the exposure to different environments. Additionally, our data are in line with previous studies performed in rats that indicated that the response to exposure to spatial exploration paradigms involved activation of new hippocampal neurons analysed 30 and 45 days after BrdU-labelling (i.e., [13, 35, 36]).

### 4.3. Expression of Arc in Newborn Cells in the Dentate Gyrus of Adult Mice after Forced-Swim Stress

Newborn hippocampal neurons are involved in the adaptation to complex and stressful environments, particularly because newborn neurons can help buffer against the effects of stress (i.e., [9, 10]). Interestingly, we found that mice exposed to forced-swim stress showed significant increase activation of newborn cells analysed six weeks post-CldU labelling (CldU/Arc) concomitantly to a significant decrease in the activation of newborn cells analysed three weeks post-IdU labelling (IdU/Arc). These results strongly suggest that the swimming performed by mice during the forced-swim task may include the activation of a neuronal ensemble that involves cells of long survival here identified with CldU labelling. Interestingly, our results are in line to what was previously reported for mice that were exposed to cold forced-swim stress [17]. In that study, the authors found a decrease in the activation of newborn cells in mice housed in voluntary physical activity conditions but not in mice housed without the physical activity stimuli. Here, it is important to note that among the differences found between that study and ours is an important aspect related to the age of the cells analysed. In our study, the newborn cells were analysed three (IdU) or six (CldU) weeks postlabelling, whereas in the previous study, the newborn cells analysed may correspond to cells ranging between 28 and 42 days postcumulative BrdU labelling [17]. Also, we found that cells analysed three weeks post-IdU labelling show decreased activation in mice exposed to forced-swim stress and similar results were recently reported with restrained stress that reduces levels of zif268, another IEG, in immature neurons but not in mature neurons. The newborn cells analysed in that study may correspond to cells ranging between 18 and 30 days postcumulative BrdU labelling [37].

## 5. Conclusion

Finally, it is important to note that we did not perform the identification of a third population of newborn neurons (CldU/IdU-cells); however, it has been reported that the number of these cells is very low [38], and it remains to be determined in the context of the paradigms used in our study. However, our data confirmed that long-survival newborn neurons in the DG, here cells analysed 42 days postlabelling, mainly respond to a paradigm including spatial exploration, as in the brief spatial exploration paradigm, in adult female Balb/C mice [11]. In addition, our study showed that cells analysed 42 days postlabelling also respond to forced-swim stress. Interestingly, previous studies suggest the possibility that newborn neurons of four or more weeks postlabelling may be preferentially recruited into circuits in the adult brain that are involved in spatial exploration and memory formation events [7–15, 36] but also after acute stress [17]. Moreover, a decrease in the activation of cells analysed 21 days postlabelling was also observed in adult female Balb/C mice (Figure 6). Thus, our study adds information related to the enrolment of newborn neurons of the DG into the neural representation of different environmental stimuli, enrolment that *may be* dependent on the age of the newborn neurons and the type of environment.

## Data Availability

The data used to support the findings of this study are included within the article.
